# A Targeted Health Risk Assessment Following the *Deepwater Horizon* Oil Spill: Polycyclic Aromatic Hydrocarbon Exposure in Vietnamese-American Shrimp Consumers

**DOI:** 10.1289/ehp.1408684

**Published:** 2014-10-21

**Authors:** Mark J. Wilson, Scott Frickel, Daniel Nguyen, Tap Bui, Stephen Echsner, Bridget R. Simon, Jessi L. Howard, Kent Miller, Jeffrey K. Wickliffe

**Affiliations:** 1 Department of Global Environmental Health Sciences, School of Public Health and Tropical Medicine, Tulane University, New Orleans, Louisiana, USA; 2 Department of Sociology, Washington State University, Pullman, Washington, USA; 3 Mary Queen of Vietnam Community Development Corporation Inc., New Orleans, Louisiana, USA; 4 The Social and Economic Sciences Research Center, Washington State University, Pullman, Washington, USA

## Abstract

Background: The *Deepwater Horizon* oil spill of 2010 prompted concern about health risks among seafood consumers exposed to polycyclic aromatic hydrocarbons (PAHs) via consumption of contaminated seafood.

Objective: The objective of this study was to conduct population-specific probabilistic health risk assessments based on consumption of locally harvested white shrimp (*Litopenaeus setiferus*) among Vietnamese Americans in southeast Louisiana.

Methods: We conducted a survey of Vietnamese Americans in southeast Louisiana to evaluate shrimp consumption, preparation methods, and body weight among shrimp consumers in the disaster-impacted region. We also collected and chemically analyzed locally harvested white shrimp for 81 individual PAHs. We combined the PAH levels (with accepted reference doses) found in the shrimp with the survey data to conduct Monte Carlo simulations for probabilistic noncancer health risk assessments. We also conducted probabilistic cancer risk assessments using relative potency factors (RPFs) to estimate cancer risks from the intake of PAHs from white shrimp.

Results: Monte Carlo simulations were used to generate hazard quotient distributions for noncancer health risks, reported as mean ± SD, for naphthalene (1.8 × 10^–4^ ± 3.3 × 10^–4^), fluorene (2.4 × 10^–5^ ± 3.3 × 10^–5^), anthracene (3.9 × 10^–6^ ± 5.4 × 10^–6^), pyrene (3.2 × 10^–5^ ± 4.3 × 10^–5^), and fluoranthene (1.8 × 10^–4^ ± 3.3 × 10^–4^). A cancer risk distribution, based on RPF-adjusted PAH intake, was also generated (2.4 × 10^–7^ ± 3.9 × 10^–7^).

Conclusions: The risk assessment results show no acute health risks or excess cancer risk associated with consumption of shrimp containing the levels of PAHs detected in our study, even among frequent shrimp consumers.

Citation: Wilson MJ, Frickel S, Nguyen D, Bui T, Echsner S, Simon BR, Howard JL, Miller K, Wickliffe JK. 2015. A targeted health risk assessment following the *Deepwater Horizon* Oil Spill: polycyclic aromatic hydrocarbon exposure in Vietnamese-American shrimp consumers. Environ Health Perspect 123:152–159; http://dx.doi.org/10.1289/ehp.1408684

## Introduction

On 20 April 2010, a large explosion on the *Deepwater Horizon* (DWH) drilling rig initiated nearly 3 months of continuous petroleum and chemical contamination flow into the waters of the Gulf of Mexico. By 15 July 2010, approximately 205 million gallons of crude oil had entered the northern Gulf of Mexico [Griffiths 2012; McNutt et al. 2012; U.S. Coast Guard (USCG) 2011]. Efforts to dissipate the oil resulted in the environmental application of approximately 2 million gallons of chemical dispersants (USCG 2011). The catastrophe spurred widespread concern about health risks associated with consumption of spill-related contaminated seafood. Concern was driven by many factors, including the state and federal closure of fisheries from May through late August 2010 in the areas under study due to the actual or anticipated presence of oil, the extensive media coverage coupled with high levels of scientific uncertainty, and potential impacts on marine ecosystems, the coastal economy, and human health. In addition, public worry regarding the ecological and human health impacts of the spill was often exacerbated by conjecture, biased information, and misinformation, as well as a lack of information and debate among experts. Knowledge gaps were often reinforced or erroneously filled by commercial and social media (Alexander-Bloch 2012; Dhar 2012). Within impacted Gulf coastal communities, such dynamics contributed to a substantial lack of trust in information from industry and government agencies. This distrust fed claims from various quarters that the risk assessment process employed by federal and state regulatory agencies in the Gulf oil spill disaster was neither transparent nor inclusive of specific community concerns.

Many members of the Vietnamese-American communities in the Gulf Coast region are directly involved in the seafood industry. Many, if not most members of this community have historically been or are now actively engaged in commercial shrimping or fishing as their primary source of income. Vietnamese Americans comprise 1.9%, 4.4%, 1.1%, 1.4%, and 3.0% of the total populations of Louisiana, Texas, Mississippi, Alabama, and Florida, respectively (Hoeffel et al. 2012). Shrimp are not only of great economic importance to Gulf Coast Vietnamese Americans, but they are also the principal seafood type consumed by this group as well as many other coastal populations. Thus, the DWH oil spill greatly impacted the Vietnamese-American community by affecting their economic stability and potentially increasing health risks associated with consuming petroleum-contaminated shrimp.

Federal government agencies, including the U.S. Food and Drug Administration (FDA) and the National Oceanic and Atmospheric Association (NOAA), were lead organizations tasked with reopening commercial fisheries in the oil-affected areas (FDA 2010). This process involved a stepwise screening of finfish, shrimp, crab, and oysters in order to determine when a fishery would be deemed safe to reopen. The first step, organoleptic testing, required the expertise of trained chemosensory testers. Organoleptic testing identified, through scent, any residual petroleum taint in seafood that may render it unsafe for human consumption. Failure to pass organoleptic screening resulted in continued fishery closures. If no petroleum scent was detected, the seafood was subjected to further testing by chemical analysis for specific oil toxicants including polycyclic aromatic hydrocarbons (PAHs). PAHs are assumed to be the primary toxicants in crude oil that may contaminate seafood, which might then pose a health risk when consumed. If the levels of 13 of the 16 priority PAHs used in health risk assessments did not reach or exceed FDA-determined consumption levels of concern (LOCs), the fisheries were reopened (FDA 2010).

LOCs for anthracene, phenanthrene, fluoranthene, fluorene, naphthalene, and pyrene—PAHs that are not considered carcinogens—were calculated using a safety threshold known as a reference dose (RfD) (FDA 2010). An RfD is based on an assumed daily exposure that does not pose a significant noncancer risk to health over an entire lifetime. LOCs for the seven priority PAHs that are considered carcinogens (7 cPAHs) {benzo[*a*]anthracene, chrysene, benzo[*b*]fluoranthene, benzo[*k*]fluoranthene, benzo[*a*]pyrene (BaP), indeno[1,2,3-*c,d*]pyrene, and dibenzo[*a,h*]anthracene} were calculated using a relative potency factor (RPF) approach, as described by the U.S. Environmental Protection Agency (U.S. EPA 1993b). Under this protocol, shrimp consumption was assumed to be 13 g of shrimp/day, which was taken from the 90th percentile of the National Health and Nutrition Examination Survey (NHANES) (Centers for Disease Control and Prevention 2003), and body weight was assumed to be 80 kg. An acceptable risk level of 1 × 10^–5^ was used as the basis for the FDA and NOAA cancer risk LOC calculation (FDA 2010). Life span, or the cancer risk averaging time, was assumed to be 78 years, and the exposure duration was assumed to be 5 years (FDA 2010). The 90th percentile NHANES seafood consumption rate and standard body weight assumptions used in the FDA health risk advisory and reopening guidelines are likely protective for the vast majority of Americans, but by definition they exclude the highest 10% of seafood consumers. Because this subpopulation has the highest exposure potential, some have argued that it should be the primary target for risk assessments (Rotkin-Ellman et al. 2012).

There has been ongoing debate in the scientific literature and media regarding the FDA’s parameter assumptions for seafood consumers and apparent exclusion of sensitive subgroups (Rotkin-Ellman et al. 2012). Our data confirm that the Vietnamese-American population in eastern New Orleans, Louisiana, represents a particularly vulnerable subgroup that eats substantially more shrimp than the NHANES estimates. In addition, the average body weight of this population is significantly less than the standard of 80 kg.

To address these possible shortcomings, we estimated health risks following both deterministic and probabilistic approaches using chemical analysis of locally harvested shrimp and household survey data collected from a random sample of adult Vietnamese Americans who worked in the local seafood industry. The research design for our study included members of this community in the entire process—from problem formulation to sample collection and communication of risk assessment results following a community-based participatory research (CBPR) model (Brown et al. 2012). To our knowledge, our study is the only DWH-related study published to date to use community-specific data, including shrimp-consumption habits and body weights, to parameterize the risk models along with chemical analyses of white shrimp (*Litopenaeus setiferus*) collected from sites that commercial shrimpers in this community traditionally use. Probabilistic risk modeling using Monte Carlo simulations of community-specific data also allowed us to address issues of uncertainty and variability not discussed in previous risk-related studies regarding the DWH event. Using this probabilistic framework, we also explored modeled consumption health risks with increasing numbers of PAH analytes to more fully explore and account for where and under what circumstances unacceptable risks may exist (Wickliffe et al. 2014). Some of our assumptions, particularly those regarding multiple PAH carcinogenicity, have not yet been scientifically investigated. However, we speculate that concerns regarding health risks from the increasing numbers of PAH analytes will be raised in future research, including effects of PAHs from oil spills on marine environments and seafood safety. Here, we present the results of our community-specific risk analysis and characterization and details of our CBPR-designed approach.

## Methods

Our study design was tailored around a community-based approach, which began with a series of discussions with community organizers, shrimpers, and the larger Vietnamese community. These meetings were ongoing through the summer and early fall of 2010 and were used to determine the objective of our study, comparative research design, and methods for harvesting and sample preparation, and to identify and involve the assistance of six community investigators. The decision to focus on Gulf white shrimp—the primary type of seafood consumed by the Vietnamese-American population in eastern New Orleans—emerged from a large community meeting with > 50 residents in attendance. Sites for sample collection were worked out among a smaller core group involving two of the authors (J.K.W. and S.F.) and six community investigators. These sites were chosen specifically because they are where shrimpers from this community have traditionally harvested shrimp. This was deemed important because the community members were most likely to consume locally caught shrimp.

*Sample collection.* White shrimp were collected from two areas: an inland area (Bayou Bienvenue) that was not oil impacted (not closed), and offshore from an area in the Chandeleur Sound that had been oil impacted and closed, but samples were collected after the fishery had been officially reopened. For the inshore area, samples from two nettings were collected on 11 November 2010 along a 2-km transect (29.988096, –89.931829; 29.996385, –89.917538) by a commercial shrimping vessel equipped with a skimmer. For the offshore area, samples from six nettings were collected by a commercial shrimp trawler on 17 November 2010 along a 25-km transect (30.044133, –89.077835 to 29.833496, –89.125214). Randomly selected shrimp (*n* = 5–10) from each netting were batched, immediately wrapped in aluminum foil, placed inside a plastic freezer bag, and iced. Five to 10 batches were collected from each netting. Shrimp were received in the laboratory within 12–24 hr of collection and transferred to a –80°C freezer for storage until analysis. Shrimp tissue samples consisting of tail muscle (abdomen) without shell were composited to yield a minimum of 20 g of material for chemical analysis. Multiple composites (*n* = 3) from each netting were sent for analysis.

*Chemical analysis*. We used quantitative PAH analysis to determine the quantities of 81 individual PAHs in extracts of shrimp abdominal tissue (see Supplemental Material, “List of all PAH analytes included in chemical analysis”). PAH analysis was performed using gas chromatography/mass spectrometry (GC/MS) in selected ion monitoring (SIM) mode. Method detection limits for this method were extremely low (< 10 ng PAH/g wet weight tissue).

The GC was temperature programmed and operated in splitless mode, and carrier flow was by electronic pressure control. The capillary column was a J&W DB-5MS^©^ (60 m long by 0.25 mm i.d. and 0.25 mm film thickness; Agilent Technologies) or equivalent. The data acquisition system allowed for continuous acquisition and storage of all data during analysis and was capable of displaying ion abundance versus time or scan number. A sample batch was analyzed as an analytical set including samples along with the following specified quality control samples: method-blank, matrix-spike, duplicate, matrix-spike duplicate, and standard reference material. A calibration curve was established by analyzing five individual calibration standards (analyte concentrations ranging from 0.02 to 1 mg/mL). For each analyte of interest, an individual relative response factor (RRF) was determined at all five calibration levels, and a mean RRF was calculated for the analyte. Calibration check standards were interspersed throughout an analytical batch to insure the instrument’s integrity. A diluted oil standard was used as a retention index solution for compounds not found in the calibration solution. Analyte concentrations were determined using the internal standard method, and analyte concentrations were corrected for surrogate recovery. Analyses were performed by TDI-Brooks International (College Station, TX, USA).

*Community survey*. To better understand how the DHW impacted shrimp consumption patterns and potential health risks among shrimp consumers in southeastern Louisiana, we surveyed Vietnamese-American adults working in the seafood industry, primarily in the shrimping sector. The Vietnamese Community Seafood Consumption Survey is a telephone and online survey designed by the research team (including community participants) in collaboration with staff from the Social and Economic Sciences Research Center (SESRC) at Washington State University. The Washington State University Office of Research Assurances determined that the study design satisfied the criteria for Exempt Research [Basic HHS Policy for Protection of Human Research Subjects; 45CFR46.101(b)(2)], and the SESRC implemented the survey in April through June 2012.

The sampling frame consisted of 375 Vietnamese adult men and women (including members in the same households) who had sought assistance from the Mary Queen of Vietnam Community Development Corporation (MQVN-CDC) in filing claims for economic losses in the weeks and months after the DWH. The MQVN-CDC provided our research team with a list of names and contact information for individuals seeking assistance, who were most likely to have direct economic ties to the seafood industry, for example, as fishermen, shrimpers, deck hands, or dock workers. This study is not, therefore, a general population survey of the affected community, but rather a targeted survey of the most economically vulnerable segment of the affected community.

Personalized letters of notification describing the survey were printed in Vietnamese and English and sent to all potential respondents. These letters were followed by a telephone survey conducted in Vietnamese by Vietnamese-speaking interviewers conversant in the local dialect or in English, as preferred by respondents. Respondents could also opt to complete the survey online in Vietnamese or English. The data-collection process utilized Tailored Design Method (TDM) principles to maximize respondent comprehension and ease navigation with the interview (Dillman et al. 2009). All respondents gave prior informed consent in accordance with institutional review board (IRB) regulations. Respondents were not compensated for their participation.

The survey consisted of 32 items. The questions were designed to collect information about respondents’ and their families’ consumption of shrimp before, during, and after the DWH. Respondents were asked how often they and their families ate locally harvested shrimp and whether the consumed shrimp were subsistence caught or store bought. The survey also included questions about portion size, preparation methods, perceived risks associated with eating shrimp, and demographic information on respondents’ gender, age, and weight, as well as ages of other family members. We examined health risk related to shrimp consumption because shrimp was the primary seafood our study population consumed. Furthermore, the study population expressed concern over whether or not Gulf shrimp was contaminated or unhealthy after the DWH event. Overall, 115 respondents fully completed the interview and 2 respondents partially completed it. This completion resulted in a response rate of 38.9%.

Interview response data were entered directly into a database by a computer-assisted telephone interviewing program (VOXCO Interviewer; Voxco, Montréal, Canada). SESRC staff then checked the data for record accuracy and imported it into IBM SPSS Statistics for Windows (version 20.0; IBM Corporation, Armonk, NY) for generation of summary statistics for each question.

*Risk assessment model input parameter distributions*. We used the Microsoft Excel add-in @Risk, version 6.01 (Palisade Corporation, Ithaca, NY) for distribution fitting, fit testing, probabilistic risk assessments, and sensitivity analyses.

Body weight. The distribution of body weight among the respondents was modeled using normal, log-normal, and Pearson 5 distributions. Goodness-of-fit testing and fit ranking were carried out using a log-likelihood approach and Bayesian information criteria.

Intake rate. The shrimp intake rate (grams of shrimp consumed per day) was calculated for each respondent:

Shrimp intake rate = (no. of shrimp/shrimp meal) × (g shrimp/no. of shrimp) × (no. of shrimp meals/week) × (1 week/7 days). [1]

The number of shrimp consumed per meal and the number of shrimp meals per week were taken directly from the survey responses. This approach allowed for the calculation of individual intake rates that take into account the consumption patterns of each individual respondent. In addition, the mass (grams) of shrimp consumed was based on the survey responses for both the number and size of shrimp consumed per meal. Commercial size classifications for shrimp were used to generate a conversion factor to determine the number of shrimp per gram of shrimp. The frequency of shrimp consumption or intake rate was converted into shrimp consumed per day in grams using the information in Supplemental Material, Tables S1 and S2. The distribution of shrimp intake rate was based on the individual survey responses and was modeled using Weibull, log-normal, and Pearson 6 distributions. These were compared and ranked in the same manner as body weight distributions.

PAH concentrations in shrimp tissue. The concentration of individual PAHs in the shrimp samples was assumed to be log-normally distributed. Log-normal distributions, based on detected mean and SD values for each individual PAH, were used to model PAH concentrations. PAHs that were below the analytic limit of detection (LOD) were assumed to be present at the LOD for each analyte divided by the square root of two.

Exposure duration. The exposure duration was assigned a uniform distribution with a range of 5–10 years. We used this distribution as a parameter in the probabilistic cancer health risk assessments. The noncancer health risk assessment process did not include the 5- to 10-year exposure duration, but instead assumed a continuous exposure over a lifetime (78 years).

*Risk assessment*. Noncancer health risk. First, we used the reference dose method to perform a noncancer risk assessment using the chemical analysis data and consumption and body weight parameters from the survey.These data were used to generate the average daily PAH intake from shrimp consumption:

Average daily intake = (PAH*_i_* × *IR*)/(*BW* × *CF*), [2]

where PAH*_i_* is the concentration in parts per million (milligrams of an individual PAH per kilogram of shrimp), *IR* is the average shrimp intake rate (Equation 1), *BW* is the average body weight from the survey data, and *CF* is a conversion factor (1,000 g shrimp/kg shrimp).

Noncancer health risk was expressed as a hazard quotient:

Hazard quotient = Daily intake/RfD. [3]

Hazard quotients were calculated as the mean and the 95th percentile of the daily intake distribution generated by a Monte Carlo analysis based on the distributions of the input variables for 10,000 iterations. Hazard quotients > 1 indicate that the specific PAH or PAHs for the same noncancer effect may pose an unreasonable health risk. PAHs with accepted U.S. EPA RfDs were included in the noncancer risk assessment: naphthalene, fluorene, anthracene/phenanthrene, pyrene, and fluoranthene (U.S. EPA 1990a, 1990c, 1991, 1993a, 1998). Alkylated homologs were assumed to have the same toxicity as the parent PAH and were included in the total PAH concentration.

Cancer health risk. To provide a conservative, quantitative measure for estimating cancer health risks due to PAH exposure, we assigned a value of LOD divided by the square root of 2 to each cPAH with an accepted RPF.

We used the U.S. EPA RPFs to calculate the BaP equivalent–adjusted cPAH concentrations in the shrimp samples (see Supplemental Material, Table S3). The total BaP equivalent concentration was calculated as the sum of the RPF-adjusted concentrations of each cPAH in micrograms per kilogram shrimp. No cPAHs were detected in any of our shrimp samples (the MDL for each cPAH was < 10 ng/g shrimp). The calculated BaP equivalent value was based on the assumption that these PAHs were present at LOD divided by the square root of 2. For the 7 cPAHs, the calculated value is 0.668 μg BaP equivalents/kg shrimp.

Monte Carlo simulations were performed using the distributions generated from the survey data as model parameters and assuming that the BaP equivalent concentrations were present in our shrimp. We calculated cancer risk over a 78-year life span (28,470 days) using Equation 4 as a basis:

Risk = {[(mg BaP_eq_/kg shrimp) × (kg shrimp consumed/day) × (365 days/year) × years exposed] ÷ (kg body weight × 28,470 days)} × oral slope factor. [4]

A conservative assumption of daily exposure (365 days per year) was used.

Exposure duration was modeled as a uniform distribution ranging from 5 to 10 years. Body weight was modeled using a truncated normal distribution to avoid unrealistically low (e.g., ≤ 0 kg) or high (e.g., ≥ 250 kg) values based on the survey data. A 78-year life span was assumed for all calculations. The standard U.S. EPA oral slope factor for BaP of 7.3 per mg/kg/day was used (U.S. EPA 1994). We used a total of 10,000 simulations to generate the cancer risk output distributions. Three iterations of risk distributions were calculated using the LOD divided by the square root of 2 value of the 7 cPAHs followed by the addition of RPF-adjusted unalkylated and alkylated PAHs as a basis.

## Results

**Figure 1:**
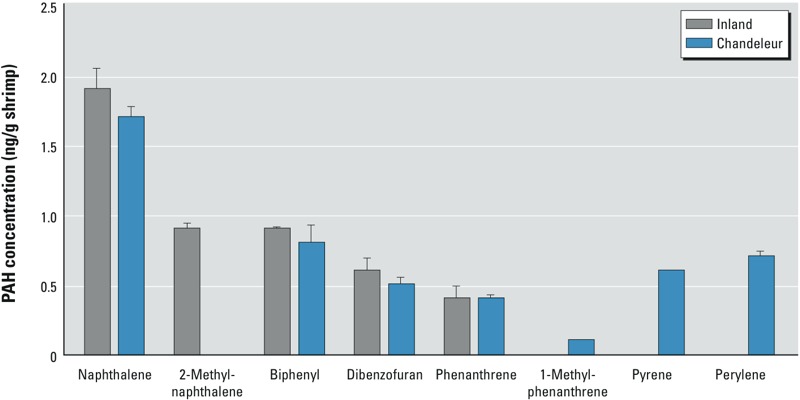
Unsubstituted and alkylated PAHs (mean and 95% confidence interval) detected in shrimp samples. Shrimp collected inland were from Bayou Bienvenue, and Chandeleur Bay is offshore.

*Chemical analysis*. Eight PAHs, including alkylated and unalkylated forms, were present at or above the MDL (quantitation limit range, 1–10 ng of a specific PAH/g of shrimp tissue). Six PAHs were detected: Naphthalene is classified as possibly carcinogenic to humans (IARC 2002; U.S. EPA 1998); biphenyl is classified as having suggestive evidence of carcinogenicity (U.S. EPA 2013); dibenzofuran, phenanthrene, and pyrene are classified as group D “not classifiable as to human carcinogenicity” (U.S. EPA 1990b, 1990d, 1991); and pyrelene is classified as IARC Group 3 “not classifiable as to its carcinogenicity to humans” (IARC 1987). Two specific alkylated PAHs (Figure 1), 2-methylnaphthalene and 1-methylphenanthrene, were found at ≥ MDL. No currently accepted RPFs are available for these chemicals.

We observed no statistically significant main effect of inland versus offshore location by two-way analysis of variance (*p* > 0.5). Further analysis by Bonferroni post hoc tests revealed small but significant differences among the individual PAHs. Naphthalene (*p* < 0.01) and 2-methylnapthalene (*p* < 0.001) were significantly higher in the inland samples; biphenyl, dibenzofuran, phenanthrene, and 1-methylphenanthrene were not significantly different between the two locations (*p* > 0.05); and pyrene (*p* < 0.001) and perylene (*p* < 0.001) were significantly higher in the offshore samples. The magnitude of all detected PAHs was very small, < 2 ppb, so care must be taken when ascribing biological meaning to these very small but statistically significant differences. We chose to combine the inland and offshore data to represent the shrimp that could be potentially consumed by our study population and to include more detected PAHs in our health risk assessments.

**Figure 2:**
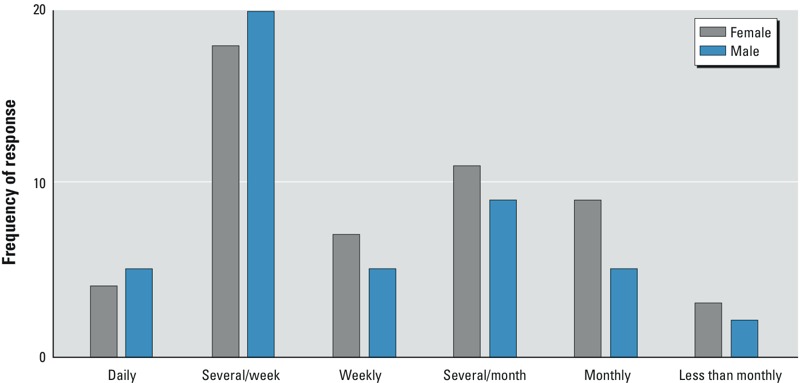
Frequency of shrimp consumption among survey respondents.

*Findings from the community survey*. The overall survey response rate was 38.9% (*n* = 115 completed surveys). Based on the Vietnamese Community Seafood Consumption Survey, the average body weight in the survey population was 63 kg (67.5 kg for males and 58.9 kg for females) (see Supplemental Material, Figure S1). The estimated average shrimp intake rate was 45.2 g/day (95% confidence interval, 12.32 g/day); the most common cooking method was boiling (see Supplemental Material, Figure S2); and the preferred size range of shrimp was medium to large (see Supplemental Material, Figure S3). The most frequently consumed meal size was 10–15 shrimp (see Supplemental Material, Figure S4), and most respondents reported eating shrimp several times per week (Figure 2).

*Noncancer risk assessment*. We calculated hazard quotients using the arithmetic mean and 95th percentile consumption rates (Table 1). Levels of PAHs in the shrimp samples were not anticipated to correspond with increased health risk after consumption, even at the 95th percentile estimate of consumption.

**Table 1:** Noncancer health risk hazard quotients (HQ) for respondents with mean and 95th percentile consumption rates.

Chemical	RfD (mg/kg-day)	Level detected (ppm)	Mean consumption HQ	95th percentile consumption HQ
Naphthalene	0.02^*a*^	4.9 × 10^–3^	1.7 × 10^–4^	2.2 × 10^–4^
Fluorene	0.04^*b*^	1.3 × 10^–3^	2.3 × 10^–5^	2.9 × 10^–5^
Anthracene/phenanthrene	0.3^*c*^	1.6 × 10^–3^	3.8 × 10^–6^	4.7 × 10^–6^
Pyrene	0.03^*d*^	1.3 × 10^–3^	3.1 × 10^–5^	3.8 × 10^–5^
Fluoranthene	0.04^*e*^	1.0 × 10^–4^	1.8 × 10^–6^	2.2 × 10^–6^
^***a***^U.S. EPA (1998). ^***b***^U.S. EPA (1990c). ^***c***^U.S. EPA (1990a). ^***d***^U.S. EPA (1991). ^***e***^U.S. EPA (1993a).				

**Figure 3:**
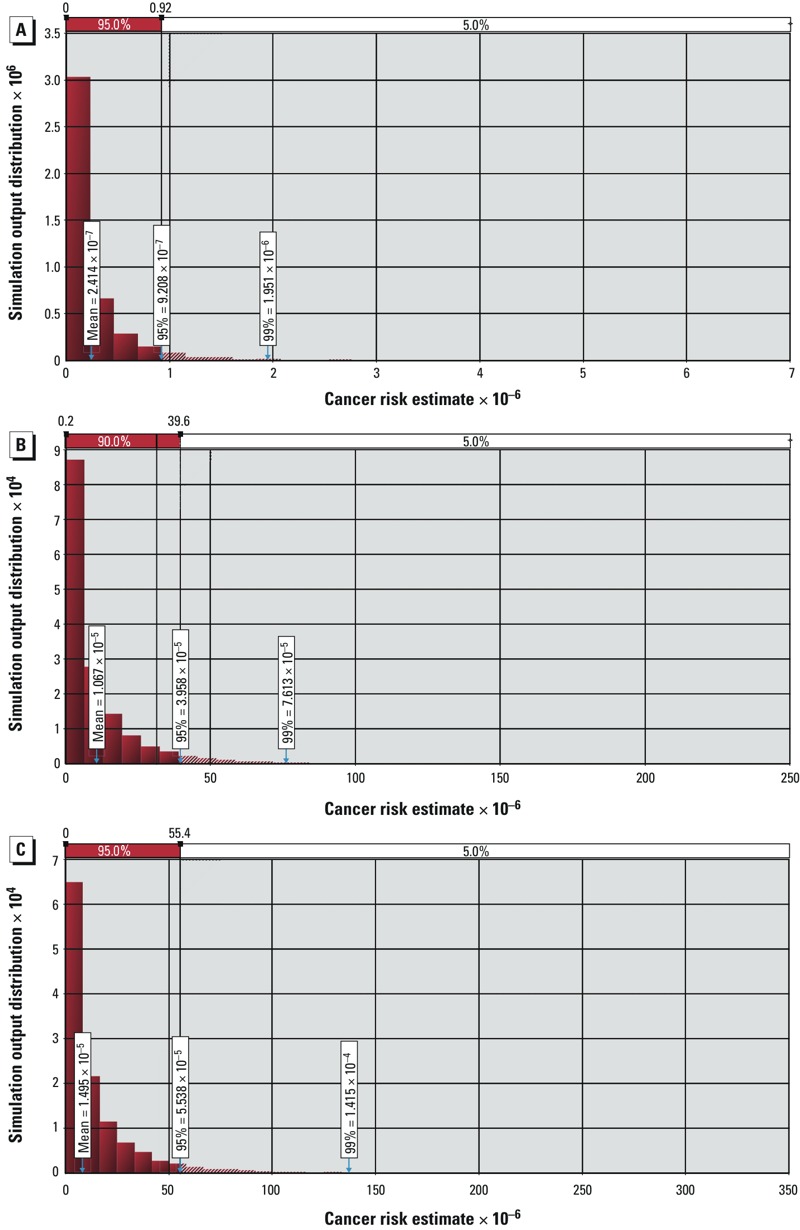
Monte Carlo simulation of cancer risk output distributions of respondents based on (*A*) 7 cPAHs, (*B*) 7 cPAHs and detected unsubstituted PAHs, and (*C*) 7 cPAHs, detected unsubstituted PAHs, and alkylated PAHs. The bars represent a relative frequency histogram of calculated cancer risks. The *x*-axis represents increasing cancer risk levels. The *y*-axis is scaled so the total height of all the bars is equal to 1 (or 100%); the *y*-axis scale varies with the magnitude of the *x*-axis so the probability density function is equal to 1. The relative frequency histogram was generated by dividing the data into intervals, counting the data points within each interval, and then dividing the counts by the total number of data points.

*Cancer risk assessment*. The modeled mean cancer risk levels from both deterministic and probabilistic models for the 7 cPAHs were an order of magnitude below a risk level of 1 × 10^–6^, including risk levels from the probabilistic risk distribution out to the 95th percentile (Figure 3A). However, when the remaining unsubstituted PAHs from the list of analytes were conservatively assigned an RPF of 1 and included in the analysis, the mean and 95th percentile risk levels met or exceeded 1 × 10^–5^ (Figure 3B). Including the alkylated PAHs, again with an assumed RPF of 1, increased the calculated mean and 95th percentile risk level by a factor of approximately 1.5 (Figure 3C). These findings show that the cancer risk estimates increased as the list of PAH analytes used for the risk calculation was expanded to include unalkylated and alkylated PAHs (Table 2).

**Table 2:** Mean, 95th percentile, and 99th percentile cancer risks with increasing number of PAH analytes.

PAH analytes	Cancer risk		
	Mean	95th percentile	99th percentile
7 cPAHs	2 × 10^–7^	9 × 10^–7^	2 × 10^–6^
7 cPAHs and unsubstituted PAHs	1 × 10^–5^	4 × 10^–5^	8 × 10^–5^
7 cPAHs, unsubstituted PAHs, and alkylated PAHs	2 × 10^–5^	6 × 10^–5^	1 × 10^–4^

## Discussion

After the DWH oil spill event, questions arose from diverse sources regarding how inclusive, and ultimately protective, the health risk assessment process used by the FDA and NOAA actually was (Rotkin-Ellman et al. 2012; Ylitalo et al. 2012). A primary concern centered on the exposure metrics used by these agencies, which some felt did not account for the substantial quantities of seafood consumed by Gulf Coast residents who lived in areas and/or worked in occupations directly affected by the oil spill (e.g., commercial shrimpers) (Rotkin-Ellman et al. 2012). The Vietnamese community in eastern New Orleans exemplifies one such community. These residents’ comparatively low body weights and high levels of seafood consumption, especially shrimp consumption, make this community particularly vulnerable. We believe that, to date, we have collected the most detailed information on DWH-related changes in shrimp consumption patterns among Vietnamese Americans in southeast Louisiana, a population whose economic and cultural ties to commercial shrimping and geographic proximity to the DHW makes them particularly vulnerable to potential hazards posed by consumption of oil-impacted shrimp.

The relatively low survey response rate (38.9%) was actually higher than expected given the specific characteristics of this “hard-to-reach” population and the unique circumstances of this community in the aftermath of the DWH event. Factors such as minority and immigrant status, lower household income and education levels, and lower English literacy can negatively affect participation in telephone surveys. (Dillman et al. 2009). Indeed 23% of the sample refused to participate, likely indicating moderate to high levels of mistrust and/or cultural insularity. Another 19% did not answer their telephones despite repeated attempts to reach them, suggesting that our sample included disconnected or nonworking lines or wrong numbers—all indicative of the highly dynamic levels of change this community was experiencing in the aftermath of the DWH. One concern with low response rates in surveys is nonresponse error, which “occurs when the people selected for the survey who do not respond are different from those who do respond in a way that is important to the study” (Dillman et al. 2009). Based on our knowledge of the target population, it is highly unlikely that responders and nonresponders are significantly different from one another in terms of shrimp preparation and consumption habits. Recall bias is another concern with survey-based data, and a limitation of our study is that it was conducted 2 years after the DWH event. However, based on the reported frequency of shrimp consumption (i.e., several times per week) within our study population, it is unlikely that recall bias would be significant. Consumption of shrimp as part of their diet is normal, and any changes from normal behavior are likely to be remembered accurately.

The seafood consumption rates used as the basis of the FDA’s LOC derivation were abstracted from the NHANES data set and do not accurately represent either the types or amounts of seafood consumed by Gulf Coast communities that may be considered vulnerable (Dickey 2012; Rotkin-Ellman et al. 2012). Our findings indicate that the shrimp consumption rate of this population is more than three times that of the NHANES 90th percentile consumption rate (44 g/day vs. 13 g/day) and that the average body weight of our respondents (63 kg) is 17 kg lower than the 80-kg standard used as the basis for the FDA risk assessment method. Although these differences in shrimp consumption rates and body weight do in fact exist, our analyses did not detect excess health risk within this Vietnamese-American community. The very low levels of PAHs detected in our cross-sectional sample of shrimp did not result in excess risk from dietary PAH exposure within our study population. Our data reflect sampling at two distinct locations on 2 different days following fishery openings approximately 6 months after the DWH event. Therefore, we cannot generalize these findings longitudinally for other dates or locations. However, the lack of risk among shrimp consumers included in our study is informative for the risk assessment process going forward.

The FDA’s guidelines for reopening fishing areas were designed to be protective of 90% of the general population; however, the remaining 10% of the population (likely the highest consumers of seafood) were specifically excluded. Therefore, the responsibility of protecting the excluded seafood consumers (i.e., consumption rate at ≥ 90th percentile) fell directly to state and local regulatory agencies (FDA 2010). We consider this approach reasonable, considering that the FDA is tasked with protecting the health of the entire U.S. population. However, it is not unreasonable to speculate that one of the many driving factors in taking this approach was the lack of relevant metrics (i.e., seafood consumption rates) among populations who fall into the upper 10% of seafood consumers.

There are limitations and assumptions in both our risk assessment methods and our analysis of our survey data. In our study, we tested only the edible tail meat from locally harvested shrimp samples. Our reasoning, informed by members of the community who participated in the research, was that shrimp was the primary seafood type consumed by our study population and that we should test the parts of the animal they actually consume. PAH content in foods can be influenced by the method used to prepare the food (Phillips 1999). Broiling, chargrilling, smoking, and blackening are all methods that increase the amount of pyrogenic—and consequently often carcinogenic—PAHs in foods. Boiling in water has little effect on PAH content, and we did determine that most of our study population consumes primarily boiled shrimp (Food and Environment Hygiene Department, Government of the Hong Kong Special Administrative Region 2004). Therefore, different cooking methods are not likely to confound our results regarding PAH content in prepared foods within our study group.

Although our study population was well balanced in terms of sex, our primary female respondents were older (see Supplemental Material, Table S4). We were therefore unable to make any type of informed statements regarding any effect the DWH spill had on the shrimp consumption patterns among women of childbearing age due to small sample size (*n* = 15). In addition, we did not capture demographic data or shrimp consumption for children. We found that primary survey respondents were likely to give information only about themselves.

The PAH levels in the seafood samples we collected and analyzed were comparable to the results of seafood analyses conducted by several regulatory agencies, academic institutions, and environmental organizations (Gohlke et al. 2011; Lubchenco et al. 2012; Rotkin-Ellman et al. 2012; Xia et al. 2012; Ylitalo et al. 2012). None of the data in the literature indicates that PAH contamination of seafood, particularly shrimp, was a serious health concern after the DWH oil spill and after reopening of the fisheries. We did find that 81% of our survey respondents reduced the amount of shrimp they consumed for at least 5 months after the DWH oil spill, and 43% reduced shrimp consumption for at least 12 months. This indicates that most of the respondents essentially conducted their own risk assessments and decided to reduce the amount of seafood they consumed without any sort of guidance from local, state, or federal regulatory agencies. We do not know what types of foods were substituted for shrimp during this period of reduced consumption or what health impacts the substitution might have had.

Many of the compounds included in our chemical analysis have no type of regulatory limits or toxicity data available. This is an emerging problem for the risk assessment process (Wickliffe et al. 2014). Some of the detected chemicals lack RPFs for even the “parent” class of chemicals. This raises the question of how to conduct health risk assessments for chemicals without regulatory guidance and/or toxicity data. Assumptions regarding a chemical’s potency or toxicity must be made for chemicals that lack regulatory standards. When assumptions are applied to compounds without adequate toxicological data in risk assessment calculations, estimates of risk will ultimately exceed acceptable risk levels. This is especially true for large classes of compounds such as PAHs. Furthermore, using an RPF approach assumes additivity across the PAHs. Thus, risk can only increase as individual PAHs are added to the risk dose model. To highlight this emerging issue, we conducted a health risk assessment where we assigned a value of LOD divided by the square root of 2 to all chemicals that were not detected. Although this is a conservative but reasonable assumption, the approach is problematic because many of these PAHs lack toxicity data and do not have RPF values. Therefore, we conservatively assigned an RPF of 1, equal to that of BaP, for all chemicals that lacked accepted RPFs. By doing so, the levels of BaP equivalents was substantially increased in our seafood samples. Consequently, as we included greater numbers of PAHs in our probabilistic analyses, risk estimates increased to what most would consider unacceptable levels. This example demonstrates the problem inherent in using an additive model to calculate health risks based on an ever-increasing list of analytes. Although this approach is clearly not ideal, the other alternative is to ignore all chemicals that do not have adequate toxicological data and accepted RPFs for risk assessment purposes, which could then lead to actual unacceptable health risks from exposure to such unstudied PAHs.

## Conclusions

Ultimately, there is an association between people with direct ties to commercial shrimping and comparatively high rates of shrimp consumption. This group represents stakeholders who are most likely to have the highest dietary exposures and suffer the greatest economic loss because of fishery closures and market loss due to the general public’s consumption concerns and changes in their dietary behaviors. The fact that these somewhat marginalized coastal communities are the most likely to experience both potential health and economic impacts of risk assessment and management policy illustrates why they should be targeted for inclusion in the risk assessment process. A more inclusive approach to risk assessment might begin with a focus on sensitive subpopulations (e.g., people with greater than average shrimp consumption). Finding no unacceptable risk within these groups would suggest that the rest of the general population will also have no unacceptable health risks. Such an approach would protect not only the general population but also would mitigate the criticism that federal policy–based risk assessment practices ignore the safety of sensitive subpopulations. Our data demonstrate that the standard exposure assumptions used as the basis for policy-based health risk assessment and the development of LOCs is not representative of shrimp consumption along the U.S. Gulf Coast. However, given that actual data from these populations was not available, it is unreasonable to hold the regulatory risk assessment process accountable for ignoring potential health effects within this vulnerable demographic.

How, then, is it possible to navigate risk assessment and the subsequent risk communication process within a community whose members are concerned about the safety of their food supply and both want and deserve access to chemical analysis data from their food sources? We argue that it is inappropriate to inform affected communities that—in the absence of regulatory information about the relevant chemicals—they are simply excluded from the health risk assessment process. As currently practiced in the risk assessment process, the intentional exclusion of 90th percentile seafood consumers presents further difficulties in the arena of effective risk communication among coastal communities.

## Supplemental Material

(389 KB) PDF

## References

[r1] Alexander-Bloch B. (2012). 2 Years after Gulf oil Spill, Louisiana Seafood Still Battling Negative Perception. Times-Picayune (New Orleans, LA) 19 April.. http://www.nola.com/news/gulf-oil-spill/index.ssf/2012/04/2_years_after_gulf_oil_spill_l_1.html.

[r2] Brown P Brody JG Morello-Frosch R Tovar J Zota AR Rudel RA 2012 Measuring the success of community science: the northern California Household Exposure Study. Environ Health Perspect 120 326 331; 10.1289/ehp.1103734 22147336 PMC3295345

[r3] Centers for Disease Control and Prevention. (2003). National Health and Nutrition Examination Survey Homepage.. http://www.cdc.gov/nchs/nhanes.htm.

[r4] Dhar J. (2012). Gulf Seafood Deformities Alarm Scientists.. http://www.aljazeera.com/indepth/features/2012/04/201241682318260912.html.

[r5] Dickey RW 2012 FDA Risk Assessment of Seafood Contamination after the BP Oil Spill [Letter]. Environ Health Perspect 120 A54 A55; 10.1289/ehp.1104539 22297092 PMC3279456

[r6] Dillman DA, Smyth JD, Christian LM. (2009). Internet, Mail, and Mixed-Mode Surveys: The Tailored Design Method. 3rd ed.

[r7] FDA (U.S. Food and Drug Administration). (2010). Protocol for Interpretation and Use of Sensory Testing and Analytical Chemistry Results for Re-Opening Oil-Impacted Areas Closed to Seafood Harvesting Due to the Deepwater Horizon Oil Spill.. http://www.fda.gov/food/ucm217601.htm.

[r8] Food and Environmental Hygiene Department, Government of the Hong Kong Special Administrative Region. (2004). Risk Assessment Studies Report No. 14: Chemical Hazard Evaluation. Polycyclic Aromatic Hydrocarbons in Barbecued Meat.. http://www.cfs.gov.hk/english/programme/programme_rafs/files/ra_pah.pdf.

[r9] Gohlke JM Doke D Tipre M Leader M Fitzgerald T 2011 A review of seafood safety after the *Deepwater Horizon* blowout. Environ Health Perspect 119 1062 1069; 10.1289/ehp.1103507 21561832 PMC3237364

[r10] Griffiths SK (2012). Oil release from Macondo well MC252 following the Deepwater Horizon accident.. Environ Sci Technol.

[r11] Hoeffel EM, Rastogi S, Kim MO, Shahid H. (2012). The Asian Population: 2010: 2010 Census Briefs.. http://www.census.gov/prod/cen2010/briefs/c2010br-11.pdf.

[r12] IARC (International Agency for Research on Cancer). (1987). Polynuclear aromatic compounds, part 1: chemical, environmental and experimental data. IARC Mongr Eval Carcinog Risk Hum 32:32–453.. http://monographs.iarc.fr/ENG/Monographs/vol1-42/mono32.pdf.

[r13] IARC (International Agency for Research on Cancer). (2002). Naphthalene. IARC Mongr Eval Carcinog Risk Hum 82:367–435.. http://monographs.iarc.fr/ENG/Monographs/vol82/mono82.pdf.

[r14] Lubchenco J, McNutt MK, Dreyfus G, Murawski SA, Kennedy DM, Anastas PT (2012). Science in support of the *Deepwater Horizon* response.. Proc Natl Acad Sci USA.

[r15] McNutt MK, Camilli R, Crone TJ, Guthrie GD, Hsieh PA, Ryerson TB (2012). Review of flow rate estimates of the *Deepwater Horizon* oil spill.. Proc Natl Acad Sci USA.

[r16] Phillips DH (1999). Polycyclic aromatic hydrocarbons in the diet.. Mutat Res.

[r17] Rotkin-Ellman M Wong KK Solomon GM 2012 Seafood contamination after the BP Gulf oil spill and risks to vulnerable populations: a critique of the FDA risk assessment. Environ Health Perspect 120 157 161; 10.1289/ehp.1103695 21990339 PMC3279436

[r18] U.S. EPA (U.S. Environmental Protection Agency). (1990a). Anthracene (CASRN 120-12-7).. http://www.epa.gov/iris/subst/0434.htm.

[r19] U.S. EPA (U.S. Environmental Protection Agency). (1990b). Dibenzofuran (CASRN 132-64-9).. http://www.epa.gov/iris/subst/0429.htm.

[r20] U.S. EPA (U.S. Environmental Protection Agency). (1990c). Flourene (CASRN 86-73-7).. http://www.epa.gov/iris/subst/0435.htm.

[r21] U.S. EPA (U.S. Environmental Protection Agency). (1990d). Phenanthrene (CASRN 85-01-8).. http://www.epa.gov/iris/subst/0459.htm.

[r22] U.S. EPA (U.S. Environmental Protection Agency). (1991). Pyrene (CASRN 129-00-0).. http://www.epa.gov/iris/subst/0445.htm.

[r23] U.S. EPA (U.S. Environmental Protection Agency). (1993a). Flouranthene (CASRN 206-44-0).. http://www.epa.gov/iris/subst/0444.htm.

[r24] U.S. EPA (U.S. Environmental Protection Agency). (1993b). Provisional Guidance for Quantative Risk Assessment of Polycyclic Aromatic Hydrocarbons.. http://www.epa.gov/oswer/riskassessment/pdf/1993_epa_600_r-93_c89.pdf.

[r25] U.S. EPA (U.S. Environmental Protection Agency). (1994). Benzo[a]pyrene (BaP) (CASRN 50-32-8).. http://www.epa.gov/iris/subst/0136.htm.

[r26] U.S. EPA (U.S. Environmental Protection Agency). (1998). Napthalene (CASRN 91-20-3).. http://www.epa.gov/iris/subst/0436.htm.

[r27] U.S. EPA (U.S. Environmental Protection Agency). (2013). Biphenyl (CASRN 92-52-4; 08/27/2013).. http://www.epa.gov/iris/subst/0013.htm.

[r28] USCG (U.S. Coast Guard). (2011). On Scene Coordinator Report: *Deepwater Horizon* Oil Spill. Washington, DC:U.S. Department of Homeland Security, U.S. Coast Guard.. http://www.uscg.mil/foia/docs/dwh/fosc_dwh_report.pdf.

[r29] Wickliffe J Overton E Frickel S Howard J Wilson M Simon B 2014 Evaluation of polycyclic aromatic hydrocarbons using analytical methods, toxicology, and risk assessment research: seafood safety after a petroleum spill as an example. Environ Health Perspect 122 6 9; 10.1289/ehp.1306724 24213154 PMC3888570

[r30] Xia K, Hagood G, Childers C, Atkins J, Rogers B, Ware L (2012). Polycyclic aromatic hydrocarbons (PAHs) in Mississippi seafood from areas affected by the Deepwater Horizon Oil Spill.. Environ Sci Technol.

[r31] Ylitalo GM, Krahn MM, Dickhoff WW, Stein JE, Walker CC, Lassitter CL (2012). Federal seafood safety response to the *Deepwater Horizon* oil spill.. Proc Natl Acad Sci USA.

