# Supplementary material for: A Targeted Health Risk Assessment Following the Deepwater Horizon Oil Spill: Polycyclic Aromatic Hydrocarbon Exposure in Vietnamese-American Shrimp Consumers
Source: Environ Health Perspect. 2014 Oct 21;123(2):152–9. doi: 10.1289/ehp.1408684 (PMC4314254; doi:10.1289/ehp.1408684)
Supplement: (389 KB) PDF [file ehp.1408684.s001.508.pdf]

# Supplemental Material

# A Targeted Health Risk Assessment Following the *Deepwater Horizon* Oil Spill: Polycyclic Aromatic Hydrocarbon Exposure in Vietnamese-American Shrimp Consumers

Mark J. Wilson, Scott Frickel, Daniel Nguyen, Tap Bui, Stephen Echsner, Bridget R. Simon, Jessi L. Howard, Kent Miller, and Jeffrey K. Wickliffe

**Figure S1.** Male and female survey respondent bodyweight.

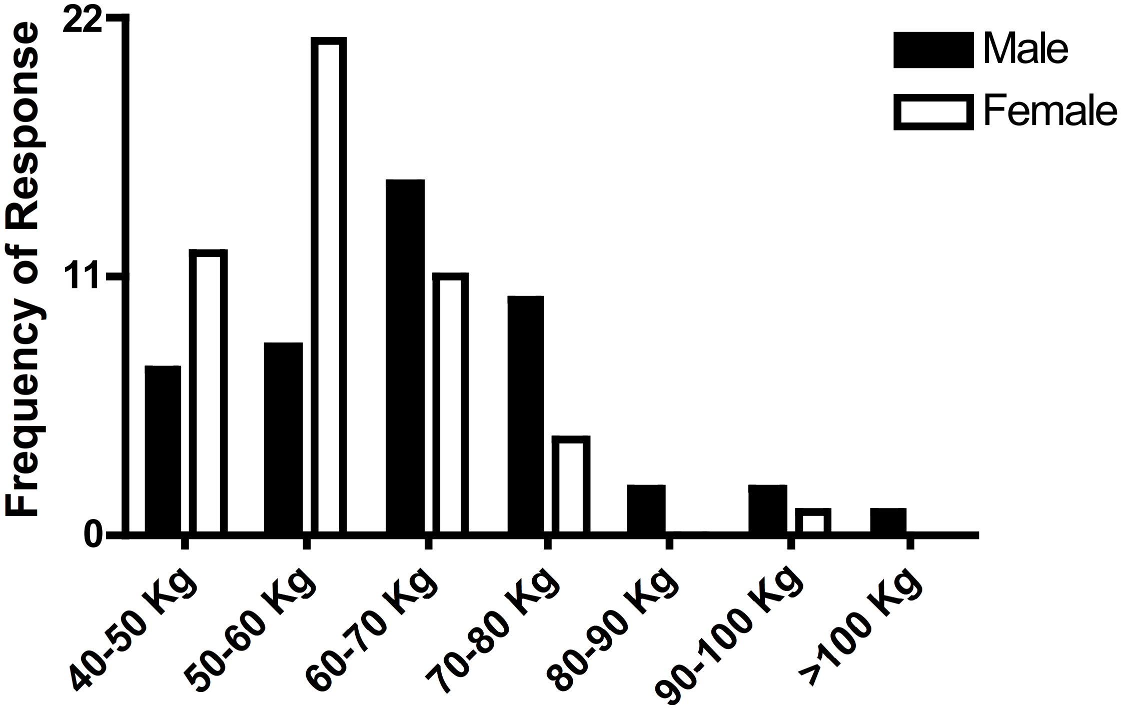

**Figure S2.** Common shrimp cooking methods among survey respondents.

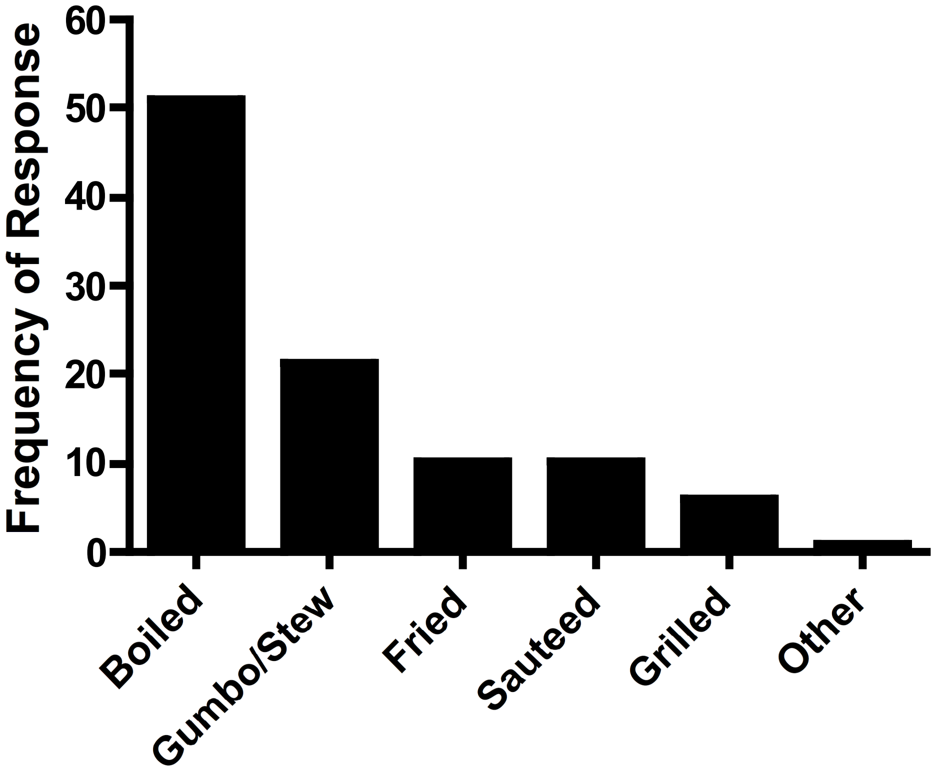

**Figure S3.** Shrimp size consumed among respondents.

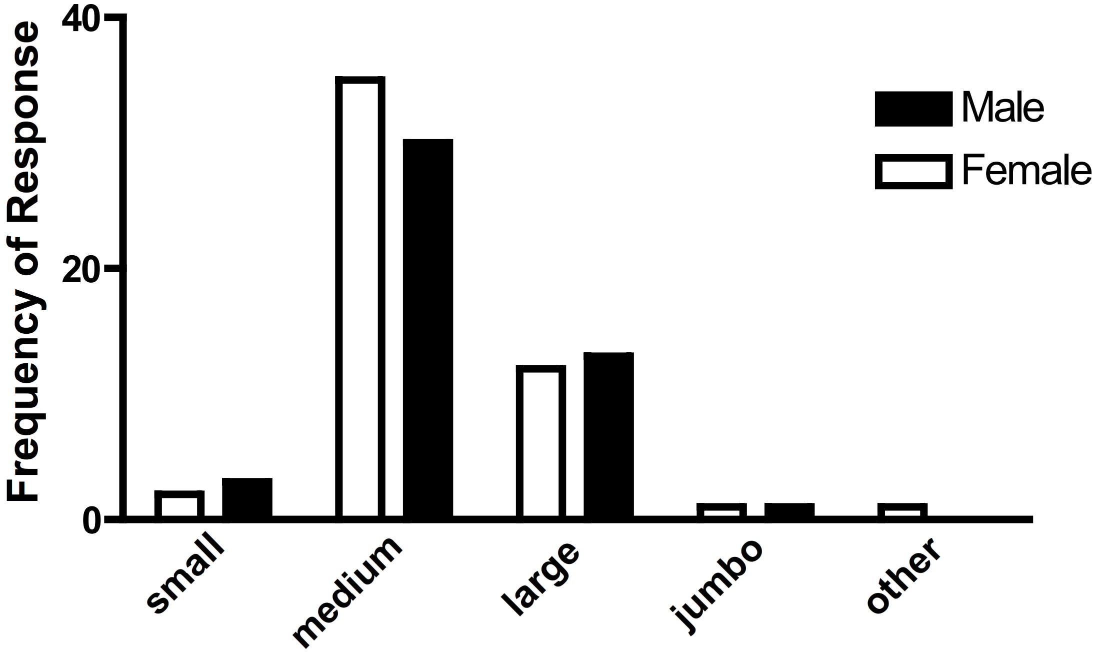

**Figure S4.** Number of shrimp consumed per meal among respondents.

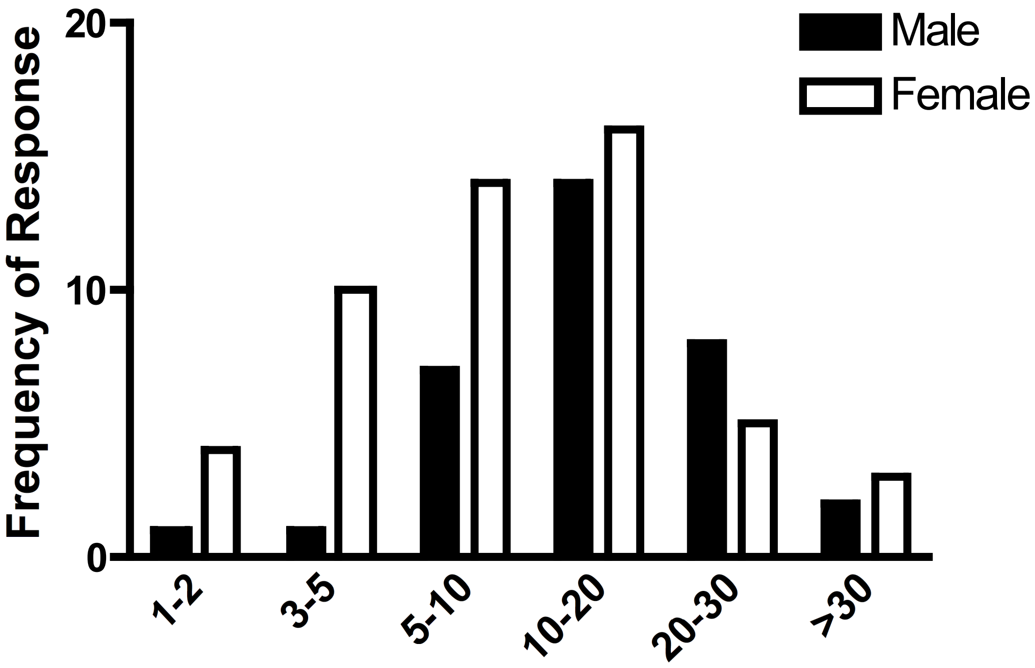

## List of all PAH analytes included in chemical analysis

Naphthalene, Dibenzofuran, Phenanthrene, Pyrene, Biphenyl, C1-Naphthalenes, Fluorene, Fluoranthene, C2-Naphthalenes, Perylene, Dibenzothiophene, Carbazole, C1-Phenanthrenes/Anthracenes, Anthracene, C1-Dibenzothiophenes, Acenaphthene, cis/trans Decalin, C1-Decalins, C2-Decalins, C3-Decalins, C4-Decalins, C3-Naphthalenes, C4-Naphthalenes, Benzothiophene, C1-Benzothiophenes, C2-Benzothiophenes, C3-Benzothiophenes, C4-Benzothiophenes, Acenaphthylene, C1-Fluorenes, C2-Fluorenes, C3-Fluorenes, C2-Phenanthrenes/Anthracenes, C3-Phenanthrenes/Anthracenes, C4-Phenanthrenes/Anthracenes, C2-Dibenzothiophenes, C3-Dibenzothiophenes, C4-Dibenzothiophenes, C1-Fluoranthenes/Pyrenes, C2-Fluoranthenes/Pyrenes, C3-Fluoranthenes/Pyrenes, C4-Fluoranthenes/Pyrenes, Naphthobenzothiophene, C1-Naphthobenzothiophenes, C2-Naphthobenzothiophenes, C3-Naphthobenzothiophenes, C4-Naphthobenzothiophenes, Benz(a)anthracene, Chrysene/Triphenylene, C1-Chrysenes, C2-Chrysenes, C3-Chrysenes, C4-Chrysenes, Benzo(b)fluoranthene, Benzo(k,j)fluoranthene, Benzo(a)fluoranthene, Benzo(e)pyrene, Benzo(a)pyrene, Indeno(1,2,3-c,d)pyrene, Dibenzo(a,h)anthracene, Benzo(g,h,i)perylene, 2-Methylnaphthalene, 1-Methylnaphthalene, 2,6-Dimethylnaphthalene, 3-Methylphenanthrene, 2/4-Methylphenanthrene, 9-Methylphenanthrene, 1-Methylphenanthrene, 4-Methyldibenzothiophene, 1-Methyldibenzothiophene, 1,6,7-Trimethylnaphthalene, 1-Methylfluorene, 2/3-Methyldibenzothiophene, 2-Methylanthracene, 3,6-Dimethylphenanthrene, Retene, 2-Methylfluoranthene, Benzo(b)fluorine, C29-Hopane,18a-Oleanane, C30-Hopane

**Table S1.** Commercial size classifications of shrimp and corresponding weight ranges.

| Size | Number of shrimp/lb | Number of shrimp/kg | Number of shrimp/g |
|---|---|---|---|
| Small | 50-60 | 110-132 | 0.110-0.132 |
| Medium | 42-50 | 92-110 | 0.092-0.110 |
| Large | 30-35 | 66-77 | 0.066-0.077 |
| Jumbo | 20-25 | 44-55 | 0.044-0.055 |

**Table S2.** Consumption frequency categories among survey respondents.

| **Reported consumption** | **# Shrimp meals/ 30 days (1 month)** |
|---|---|
| Daily shrimp meal | 30 shrimp meals/30 days |
| Several shrimp meals/week | 14 shrimp meals/30 days |
| One shrimp meal/week | 4 shrimp meals/30 days |
| Several shrimp meals/month | 2 shrimp meals/30 days |
| One shrimp meal/month | 1 shrimp meal/30 days |
| Less than one shrimp meal/month | 0.5 shrimp meals/30 days |

**Table S3.** Relative potency factors for carcinogenic PAHs used by the USEPA and the USFDA for health risk assessments. Benzo[a]pyrene is considered the index PAH.

| **PAH** | **Relative potency factors for cPAHs[a]** |
|---|---|
| Benzo[a]anthracene | 0.1 |
| Chrysene | 0.001 |
| Benzo[b]fluoranthene | 0.1 |
| Benzo[k]fluoranthene | 0.01 |
| Benzo[a]pyrene | 1 |
| Indeno[1,2,3-c,d]pyrene | 0.1 |
| Dibenzo[a,h]anthracene | 1 |

[a]Collins et al. 1998; USEPA 1993.

**Table S4.** Respondent demographics.

| Characteristic | Males | Females | Females of child bearing age |
|---|---|---|---|
| Average age | 49.4 years | 50.5 years | 34.5 years |
| Age range | 28-76 years | 26-74 years | 26-43 years |
| Average bodyweight | 67.5 kg | 58.9 kg | 57.8 kg |
| Body weight range | 45-113 kg | 40-92 kg | 40-72.5 kg |
| Number of respondents | 49 | 64 | 15 |

## References

Collins JF, Brown JP, Alexeeff GV, Salmon AG. 1998. Potency equivalency factors for some polycyclic aromatic hydrocarbons and polycyclic aromatic hydrocarbon derivatives. Regulatory toxicology and pharmacology : RTP 28:45-54.

USEPA. 1993. Provisional guidance for quantative risk assessment of polycyclic aromatic hydrocarbons. Washington D.C.
